# Data-driven quality improvement in low-and middle-income country health systems: lessons from seven years of implementation experience across Mozambique, Rwanda, and Zambia

**DOI:** 10.1186/s12913-017-2661-x

**Published:** 2017-12-21

**Authors:** Bradley H. Wagenaar, Lisa R. Hirschhorn, Catherine Henley, Artur Gremu, Ntazana Sindano, Roma Chilengi, the AHI PHIT Partnership Collaborative, Ahmed Hingora, Dominic Mboya, Amon Exavery, Kassimu Tani, Fatuma Manzi, Senga Pemba, James Phillips, Almamy Malick Kante, Kate Ramsey, Colin Baynes, John Koku Awoonor-Williams, Ayaga Bawah, Belinda Afriyie Nimako, Nicholas Kanlisi, Elizabeth F. Jackson, Mallory C. Sheff, Pearl Kyei, Patrick O. Asuming, Adriana Biney, Roma Chilengi, Helen Ayles, Moses Mwanza, Cindy Chirwa, Jeffrey Stringer, Mary Mulenga, Dennis Musatwe, Masoso Chisala, Michael Lemba, Wilbroad Mutale, Peter Drobac, Felix Cyamatare Rwabukwisi, Lisa R. Hirschhorn, Agnes Binagwaho, Neil Gupta, Fulgence Nkikabahizi, Anatole Manzi, Jeanine Condo, Didi Bertrand Farmer, Bethany Hedt-Gauthier, Kenneth Sherr, Fatima Cuembelo, Catherine Michel, Sarah Gimbel, Bradley Wagenaar, Catherine Henley, Marina Kariaganis, João Luis Manuel, Manuel Napua, Alusio Pio

**Affiliations:** 1 Department of Global Health, School of Public Health, University of Washington, 1959 NE Pacific Street, Seattle, WA 98195 USA; 2 Health Alliance International, Seattle, WA USA; 3 Feinberg School of Medicine, Northwestern University, Chicago, IL USA; 4 Partners in Health, Kigali, Rwanda; 5 Health Alliance International, Beira, Mozambique; 6 Centre for Infectious Disease Research in Zambia, Lusaka, Zambia; 7 University of North Carolina at Chapel Hill, Chapel Hill, NC USA

**Keywords:** Quality improvement, Low income, Health systems research, Health systems strengthening, Data assessment, Decision making, Maternal and child health, Rwanda, Mozambique, Zambia

## Abstract

**Background:**

Well-functioning health systems need to utilize data at all levels, from the provider, to local and national-level decision makers, in order to make evidence-based and needed adjustments to improve the quality of care provided. Over the last 7 years, the Doris Duke Charitable Foundation’s African Health Initiative funded health systems strengthening projects at the facility, district, and/or provincial level to improve population health. Increasing data-driven decision making was a common strategy in Mozambique, Rwanda and Zambia. This paper describes the similar and divergent approaches to increase data-driven quality of care improvements (QI) and implementation challenge and opportunities encountered in these three countries.

**Methods:**

Eight semi-structured in-depth interviews (IDIs) were administered to program staff working in each country. IDIs for this paper included principal investigators of each project, key program implementers (medically-trained support staff, data managers and statisticians, and country directors), as well as Ministry of Health counterparts. IDI data were collected through field notes; interviews were not audio recorded. Data were analyzed using thematic analysis but no systematic coding was conducted. IDIs were supplemented through donor report abstractions, a structured questionnaire, one-on-one phone calls, and email exchanges with country program leaders to clarify and expand on key themes emerging from IDIs.

**Results:**

Project successes ranged from over 450 collaborative action-plans developed, implemented, and evaluated in Mozambique, to an increase from <10% to >80% of basic clinical protocols followed in intervention facilities in rural Zambia, and a shift from a lack of awareness of health data among health system staff to collaborative ownership of data and using data to drive change in Rwanda.

**Conclusion:**

Based on common successes across the country experiences, we recommend future data-driven QI interventions begin with data quality assessments to promote that rapid health system improvement is possible, ensure confidence in available data, serve as the first step in data-driven targeted improvements, and improve staff data analysis and visualization skills. Explicit Ministry of Health collaborative engagement can ensure performance review is collaborative and internally-driven rather than viewed as an external “audit.”

**Electronic supplementary material:**

The online version of this article (10.1186/s12913-017-2661-x) contains supplementary material, which is available to authorized users.

## Background

Across most low-and middle-income countries (LMICs), health system decentralization has increased the decision-making responsibility of sub-national management teams. As the science of quality improvement (QI) has evolved, there has been an increased focus on the use of high-quality data to identify gaps in service delivery and inform improvement approaches; ensure data-driven decision making and resource allocation; and strengthen the use of evaluative designs for QI that maximize causal inference [1–4]. While “data-driven” decision-making often refers to the use of quantitative data, including routine health information systems (RHIS), chart reviews, and intermittent survey data such as Demographic and Health Surveys, evidence from LMICs suggest that district managers also utilize written, verbal, observational, experiential, and training data that exist outside formal routine and survey data sources to inform decision-making [5].

Recently there has been significant attention paid to the improvement of RHIS data quality, and a general consensus has emerged that rapid data quality assessment (DQA) approaches which incorporate improvement methods to address data quality gaps can successfully improve RHIS data quality in LMICs [6–9]. However, less is known about evidence-based interventions to subsequently improve the use of these data for decision-making around improving quality, effectiveness, and efficiency of service delivery. The accumulation of diverse high-quality health data without effective data-use cultures, support structures, capacity, and systems needed to implement, monitor, and evaluate change means that much effort is spent collecting and improving data without approaches to leverage those data for the development of health system improvements. In addition, there has been limited attention paid to how best to leverage the interconnections across all available data sources to understand and improve health service delivery.

In this paper, we define health QI as the continuous efforts of everyone interacting with the health system (healthcare workers, patients, communities, researchers, managers, educators, and policymakers) to make changes that lead to better patient outcomes, system performance, and capacity development [10]. Barriers to using data for QI occur across strategic, cultural, technical, and structural dimensions and include lack of time, inability to access data, difficulties understanding statistics or data-related language, and real or perceived inability to change practice [11]. Frequently identified barriers to the use of evidence for higher-level policymaking include: the inability to access high-quality data, followed by clarity, relevance, and reliability of research findings [12]. Previous data-use for QI models have been implemented in Tanzania [13], Kenya [14], Côte d’Ivoire [15], and South Africa [16]. Yet, most of these interventions have been demonstration projects implemented in one setting without long-term follow-up or measurement of service delivery impacts. Moreover, a number of questions remain unanswered, such as the best methods for increasing data use, and how to measure data use for decision making.

In this paper we present different approaches to improving data-driven QI that have been implemented across three countries over the past seven years as part of the Doris Duke Charitable Foundation’s African Health Initiative (AHI) [17]. We discuss the different theories of change, intervention, implementation, and measurement approaches, and discuss common and divergent lessons learned. We believe this paper is of high interest to researchers, implementers, and policymakers working to promote the use of routine and population-based data sources for health system quality gains across LMICs.

## Methods

Eight semi-structured in-depth interviews (IDIs) were administered to program staff working in each country during a writing workshop in October 2015. This workshop focused on program evaluation and sharing cross-country lessons learned and included breakout sessions and on-on-one discussions and interviews. No informed consent was collected. IDIs for this paper included principal investigators of each project, key program implementers (medically-trained support staff, data managers and statisticians, and country directors), as well as Ministry of Health counterparts. IDI data were collected through field notes; interviews were not audio recorded. Data were analyzed using thematic analysis but no systematic coding was conducted. IDIs were supplemented through donor report abstractions, a structured questionnaire, one-on-one phone calls, and email exchanges with country program leaders to clarify and expand on key themes emerging from IDIs. Consensus decision-making was used to settle on a unifying theoretical change approach for promoting data-driven QI during the in-person meetings, and was iteratively reviewed and improved during follow-up emails and phone calls with authors. Cross-country analyses were summarized by the lead author, with input from all other authors and program staff from each country. This paper describes elements of each broader AHI project’s efforts around data-driven QI. For more information on full intervention protocols, overall project motivations and goals, and implementation details, we suggest the following previous publications [18–20].

## Results

**Fig. 1:**
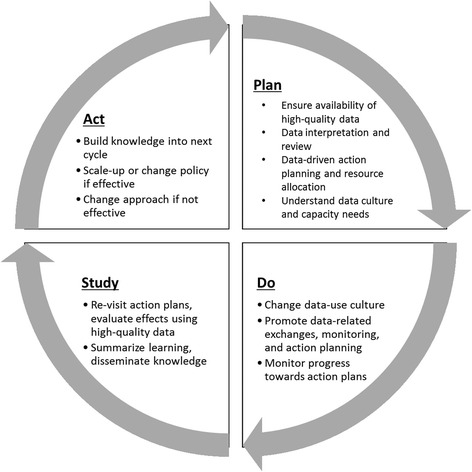
Modified Plan, Do, Study, Act framework used to inform development and implementation of data-driven QI approaches across the three study countries (Mozambique, Rwanda, Zambia)

Consensus decision-making arrived at a unifying theoretical change approach informed by the already-existing and well-known Plan-Do-Study-Act (PDSA) framework [21] to evaluate the steps in data-driven QI within each country project and support cross-project analyses. The model includes modified Plan-Do-Study-Act stages that are used iteratively for QI (see Fig. 1). Table 1 summarizes the PDSA cycle findings across each country program.

**Table 1:** Cross-site table of activities, measures of successes, common elements, and lessons learned across 7 years of implementation experience in Mozambique, Rwanda, and Zambia for promoting data-driven quality improvement

Quality improvement stage	Mozambique		Rwanda		Zambia		Common approaches	Lessons learned
	*Activities*	*Measures of success*	*Activities*	*Measures of success*	*Activities*	*Measures of success*		
*Plan*	1. Ensure high-quality routine data exist through facility-level DQA of RHIS data 2. Training in data review, analysis with systematized data matrices	1. Average routine data concordance achieved >80% 2. Demonstrated competence in visualization and analysis; ascension from stage 0 to stage VI of Berwick’s stages (see Fig. 2)	1. Mentoring on DQAs at facility and district level 2. Training on data review, quality improvement, and analysis 3. Determining best ways for performance review and feedback	1. Increased community health worker data quality [43] 2. Demonstrated use of data to drive new change concepts 3. Collaborative development of MESH program [25]; ascension from stage 0 to stage V of Berwick’s stages (see Fig. 2)	1. Collaboratively create new tools for input, visualization, and management of health data, including training in analysis/interpretation 2. Pre-implementation facility assessment around supplies, pilot DQA, and clinic support workers	1. New tools in place, operating, and staff demonstrating competence with new procedures 2. Minimum standards met as evaluated in pre-implementation assessment; ascension from stage 0 to stage V of Berwick’s stages (see Fig. 2).	1. Began activities after known gaps in data quality and use 2. DQA activities were conducted first 3. Trainings in data analysis and interpretation 4. Tools for systematized data visualization, feedback, and supervision	1. Begin with DQAs to improve analysis skills, promote that change is possible, and ensure high-quality data 2. Ensure Ministry of Health engagement and ownership to allow flexibility in health system protocols and ensure sustainability. 3. Progress to “Do” step once achieving stage IV or V on Berwick’s stages
*Do*	1. Begin district-level data review and feedback meetings 2. Create collaborative and iterative data-driven action plans 3. Supervision from provincial and district teams to follow-up action planning	1. 56 meetings conducted from 2012 to 2015 2. 498 collaborative action plans implemented and iteratively reviewed 3. Supervision conducted through project period to all, and more intensive supervision to low-performing districts and facilities	1. Integrate data-driven performance review and feedback into management meetings at facility, district, and province. 2. Implement MESH program [25] 3. Field-based operations research training and Masters in implementation science	1. Increased use of data feedback to make decisions in meetings 2. Change in performance measures across areas of focus [25] 3. Number of peer-reviewed papers published and degrees awarded	1. Basic infrastructural upgrade and equipment 2. Formally implement clinic support worker tasks 3. Use new data tools to conduct automatic patient tracking and real-time indicator monitoring. 4. Dedicated QI mentoring teams at district level provide supervision and performance review with feedback	1. Functioning equipment and EMR system to provide real-time data collection and feedback 2. Support workers successfully completing tasks 3/4. Performance indicators being tracked in real-time and reviewed during mentorship visit by QI teams	1. Used iterative performance review and feedback from within the system – collaboratively – instead of an external “audit” 2. Used supervisors working within the government system to build sustainability and engender a culture of data-use, change, and shared accountability.	1. Using Ministry of Health supervisors is essential to ensure a culture of collective improvement and performance review instead of negative feelings of external “audit” 2. Relying on external funding for advanced data technology can undermine sustainability and government ownership
*Study*	1. Re-visit action plans and performance data during next meeting 2. Conducted qualitative evaluation of meetings among 21 facility staff and 21 managers/supervisors 3. Formal evaluation forthcoming	1. Equal number of meetings and action plans each year incorporating feedback from previous cycles, along with data-driven targeting of resources and supervision 2. Need for more systematized review and support, and suggestions to conduct meetings quarterly instead of bi-annually. 3. Evaluation will be published separately Fall 2016.	1. Assess data quality improvements 2. Assess the effects of data-driven QI changes instituted 3. Assess the culture of data use	1. Observed improvements in RHIS data quality [28] 2. Observed improvements in data-driven QI [29, 30] 3. Qualitative evidence of increase in health workers’ value and ownership of data; capacity building efforts to increase use and value of programmatic data [31]	1. Indicators of data timeliness, form completeness, adherence to care protocols tracked 2. District-level QI teams conducted chart reviews to test data quality and validity 3. Monthly targets for supervision visits	1. Median time from consultation to data visible was 40 h; improvements from 8.4% blood pressure measurements to 81.5%, among others. 2/3. QI teams conducted 2.5 monthly chart review/mentoring visits per facility	1. Use performance review and feedback data to feed back into intervention to improve quality of data, quality of QI change concepts, quality of practice, management, and supervision 2. Use key tracer indicators to allow assessment of data quality yet avoid too much burden	1. Future projects should devise implementation measures and easy ways to track success or failure of action plans and build these performance measures into overall program 2. Staff turnover is major challenge which impacts continuity of data review, action planning, and QI change concepts. 3. Engaging higher-level managers and supervisors can help avoid changing higher-level priorities that can eliminate space for change at lower levels
*Act*	1. Pilot testing structured action plan monitoring tool 2. Meetings expanded to pharmacy, malaria, and tuberculosis 3. Development of new IDEA project to focus on intensive measurement and scale-up to Manica province	1. Data forthcoming on pilot test 2/3. Proposal funded by DDCF to scale-up meetings, including more in-depth monitoring and process/impact evaluation for next 5 years, including an additional 7 districts.	1. Work with district and national program to expand lessons learned on DQAs 2. Analysis of implementation needs for MESH and adaptation for national spread 3. Plans to sustain field research training, plans developed for spread to other settings	1. Ongoing DQA at district-level; routine publication on quality at national level 2. Scale-up of MESH nationally for priority areas; development of toolkit for spread to other countries 3. Spread of implementation research to other PIH-supported sites; increase in implementation research focused degrees; number of new publications and new primary authors	1. Target additional supportive supervision or resources to low performing facilities or clinicians 2. Identify data gaps and create revised tools 3. Replacement of obsolete or non-functional equipment 4. Policy-level feedback on performance of the system	1. Additional support sought from new funder to sustain intervention 2. New forms created for neonatal care, and revisions done to existing tools to enhance efficiency 3. Number of facilities sustaining all QI activities 4. Challenges identified and corrective action plans discussed with leadership	1. Iterative feedback at multiple levels of the health system 2. Continued efforts to mobilize resources in order to improve/sustain intervention	1. Continued and active engagement with the Ministry is critical 2. Ministry may not have resources to sustain entire QI intervention activities – active engagement will help identify “core” components required for sustainability of effects

**Fig. 2:**
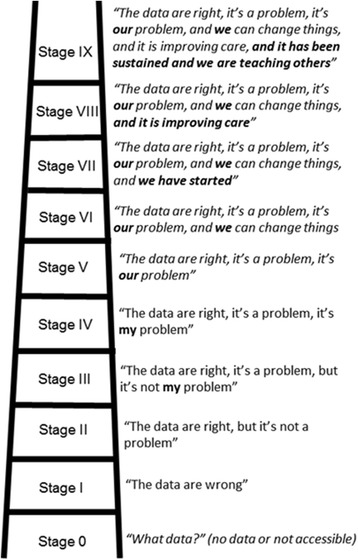
Hirschhorn Partners In Health framework for data utilization for QI stages, or “ladder”, building on Berwick’s coping with data [23]. New stages are italicized

*DQA* Data quality assessment, *RHIS* Routine Health Information System, *MESH* Mentoring and Enhanced Supervision at Health Centers; Berwick’s stages – see Fig. 2; *PIH* Partners in Health

### Step 1: “Plan”

Data use improvement approaches were developed after all teams documented gaps in both data quality and data use to drive decision-making and health system evaluation. Prior to intervention initiation, across all three projects, there was limited use of data to drive improvements in quality of care provision, supervisory activities, and in management decisions. Following this gap identification, country teams engaged in the “Plan” step to first ensure the availability of high-quality RHIS and other health system data. Work also included gaining in-depth understanding of current data-use cultures and capacity needs. Teams then spent time strategizing how to train and motivate staff around data interpretation to inform gap identification, performance monitoring/review, action planning, and resource allocation.

The “Plan” step in Mozambique involved the implementation of facility-level RHIS DQAs, along with training of sub-national managers on data analysis and the use of systematized data matrices to present secular trends in outputs and coverage estimates across a range of key health system indicators. Mozambique progressed to the “Do” step (described below) only after data concordance achieved >80% across tracer facilities (achieved in 2012) [9, 22]. Lessons from these collaborative DQAs were carried forward into the next QI steps by encouraging health staff to consider data quality issues and to apply other analysis skills such as the importance of disaggregation when interpreting data for decision-making [18].

Rwanda, by contrast, developed a theory of change progressing from lack of access to data and absence of a culture of learning, to a state of routine data utilization to identify and drive change and share successes. This model was developed from an expanded Berwick’s model of data utilization for QI [23] (see Fig. 2), and the Partners In Health (PIH) model of integrating monitoring and evaluation with QI [24]. A number of key interventions were designed to address data access, data quality, and the culture and skills needed to increase data-driven decision making for QI [22]. These included mentoring on DQAs at the facility and district level; measuring, mentoring, and performance feedback for clinicians and managers; incorporation of data feedback into district management meetings; and capacity building on monitoring and evaluation using RHIS data (see Table 1; see Additional file 1).

Zambia focused planning activities on identifying data needs to monitor the Integrated Management of Childhood Illness and the Integrated Management of Adult Illness guidelines. These standard guidelines were adapted to the Zambian context and were then used to create new tools for input, visualization, and management of health data at the district, clinic, and individual clinician levels. New tools were built in partnership with stakeholders, Ministry representatives, the study team, and health staff working at all levels. The planning stage included a pre-implementation facility assessment to target missing equipment and supplies, along with ensuring the presence of clinic support workers who maintained the new medical record systems.

### Step 2: “Do”

The “Do” phase across each of the three countries focused on implementing data-focused meetings, monitoring activities, and implementing feedback loops and other intervention activities, along with action planning. In Mozambique, the main intervention component of district performance review and enhancement meetings was formally started. During these meetings, health facility leadership use standardized data matrices to present and review secular trends in RHIS outputs and coverage estimates across a range of key indicators. With AHI team support, district and provincial managers responsible for supervisory roles for facilities developed collaborative action plans targeted at priority indicators and areas in need of most improvement. If necessary, AHI funds were available for infrastructure improvements or to support other improvement activities. From January 2012 through June 2015, a total of 140 facilities participated in performance review and enhancement meetings and developed 498 action plans across the 13 districts of Sofala Province. Detailed information on the organization of the intervention in Mozambique has previously been described elsewhere [18].

Rwanda “Do” activities were more diverse (see Table 1; see Additional file 1), yet had similar foci as Mozambique in that their core component was continual review of health system data, along with the integration of data-driven performance review and feedback into management and district meetings. The goals were similar as well – to increase the use and value of known high-quality data to identify and address gaps in management and system performance. Performance review and feedback activities at higher management levels were supplemented with the implementation of the Mentoring and Enhanced Supervision at Health Centers (MESH) program to promote integrating data use for individual clinical mentoring and clinical systems improvement. For more detailed information on the organization and structure of the MESH program, see other papers [25–27].

At the “Do” stage in Zambia, task-sharing was implemented to shift facility-level data gathering and management away from clinicians and to clinic-support workers, enabling clinicians to dedicate more time to patient care [20]. Servers were set up at the district medical office to track performance indicators in real-time, allowing rapid care improvement by monitoring patient “danger signs” to ensure follow-up of patients. For example, if patients did not return for follow-up visits, community health workers would be automatically notified via text message to visit the patient at home. Dedicated QI mentoring teams at the district level began visiting facilities regularly, targeting clinics and individual physicians based on performance indicators to discuss and address low-performing areas. Data-driven decisions were also supported at the provincial and national levels. For example, the number of cases of malaria was used for real-time resource allocation, such as the allocation of malaria test kits and reagents.

### Step 3: “Study”

During the “Study” phase, teams across sites re-visited protocols, responded to performance review/feedback, re-visited action plans, and evaluated the effects of data-driven changes using data produced by the health system. Mozambique and Rwanda continued throughout the remainder of the project to engage in performance review and feedback meetings, with subsequent meetings presenting key health data indicators and whether changes implemented through action planning had the desired effects.

In Mozambique, after a number of action-planning cycles had been completed, a preliminary qualitative evaluation of the district performance review and enhancement meetings was conducted, involving in-depth-interviews with 21 health facility staff, 14 district managers, and 7 provincial managers. These interviews highlighted positive intervention effects on: (1) timeliness of facility data submission to district managers and the effective organization/storage of data; (2) data analysis and interpretation capacities; (3) improved recognition of the importance of RHIS data by frontline health workers; (4) improved targeting of district and provincial managers to low-performing health facilities; and (5) improved sharing of new technical skills and changing policies around malaria, HIV, and tuberculosis case management. Suggestions for improvements included the need for systematized review and support (supervision and financial) between meetings to meet action-plan goals, and to conduct meetings more frequently (3 month cycles instead of 6 month).

In Rwanda, activities improved data quality in a number of key areas, and also contributed to national efforts around RHIS performance [28]. The program also saw effective data utilization to allocate resources for health systems strengthening and data feedback for performance-based financing and driving QI at the health center and district levels [25, 27, 29, 30]. A qualitative evaluation found a strong increase in health workers’ value and ownership of existing and future data. Quantitative analyses of data-driven action planning and change concepts have shown positive improvements in a number of areas [29, 30].

In Zambia, at the clinic and individual clinician level, indicators of data timeliness, form completeness and adherence to care protocols were tracked. District-level QI teams conducted chart reviews to cross-check with the electronic medical record (EMR) system and test the validity of the system. QI teams had monthly targets to meet for the number of chart reviews and mentoring/supervision visits conducted. Performance tracking efforts found: (1) positive qualitative reports from stakeholders; (2) increased adherence to protocols on pneumonia, diarrhea, malaria, and malnutrition; (3) an increase in the percentage of cases “closed”; and (4) heightened confidence in the system, measured through increased health utilization. Before and after comparisons of data completeness on vital signs (temperature, pulse, respiratory rate, and blood pressure) also showed dramatic improvements from pre-intervention levels in 2010 (see Additional file 2). As of July 2015, the median time from consultation to patient data visible in the electronic database was 40 hours. QI teams conducted a monthly average of 2.5 chart reviews and mentoring visits per facility. By June of 2015, 80.1% of pediatric cases (75,556/93,294) and 80.2% of adult cases (78,342/97,645) with danger signs were successfully tracked and closed. By July 2015, 71.6% of malaria cases were appropriately managed according to standardized protocols, along with 76.2% of pneumonia cases, and 63.5% of diarrhea cases.

### Step 4: “Act”

The “Act” phase involved building knowledge from the experience and “study” phase into the next cycle to increase data utilization for QI, feeding results back into the project designs and health system to potentially generate higher-quality data, and disseminating findings when appropriate. In addition, work to integrate and spread components which were successful was also a main target towards the later years of the projects.

Due to the ongoing success of the performance review and enhancement meetings throughout the 7-year AHI project in Mozambique [18], these meetings have become a cornerstone of the Mozambique AHI project’s efforts to improve the quality of service delivery in primary health clinics in Sofala Province. Unlike many other interventions where a main challenge is uptake, these meetings have become immensely popular with Ministry partners and spread has occurred. Meetings began in the maternal and child heath arena, but have been expanded to pharmacy, malaria, and tuberculosis, due to specific requests from program managers and higher-level decision makers. Requests were also made for further expansion into HIV; however, there have not yet been sufficient funds or staff for this expansion. The Mozambique team was also funded to scale-up these activities to nearby Manica Province and to improve process and impact evaluation of ongoing QI activities. These meetings were popular amongst Ministry partners for four reasons. First, the Provincial Ministry of Health Maternal and Child Health Directors were engaged at the outset of the process, and promoted attendance at these meetings as a key staff responsibility. Second, through the meetings, facility staff had an opportunity to travel to the largest city in each district. Third, District managers profited from the meetings to notify staff of new guidelines, protocols, or other routine supervisory discussions. Last, once other Provincial Directors saw these activities and their benefit, they sought to replicate for other programs, thus increasing the popularity of the activity.

In Rwanda, the use of data-driven QI has continued despite the decrease in direct support from the grant, showing further evidence of culture change and potential for sustainability. Data are now routinely presented and discussed at district meetings and a number of the interventions have been integrated into routine management such as performance indicators and review, and including data use for QI into the facility mentoring program. The success in MESH to use data to drive mentoring has resulted in spread to other areas of care in the supported districts, including scale-up activities for HIV and neonatal mortality reduction in Rwanda. Knowledge from the overall intervention package has been sustainably integrated into research capacity building at the national university and packaged for replication in other countries [31].

The intervention in Zambia showed positive clinical effects (see Additional file 2), but given the level of technological complexity and skills needed to maintain the EMRs, touch-screen terminals, and computers which enabled real-time performance review and feedback, it is unclear the level of sustainability offered now that the intervention is complete. Staff turnover and challenges in collaborative implementation of intervention components also limited system integration. Using the Atun et al. 2010 framework for integration of health systems innovations, this intervention ranked high on intervention complexity through multiple episodes of care, multiple intervention elements, multiple levels of the system, and necessitating high levels of user engagement for success [32].

Scale-up and spread to new areas has not occurred, although elements of the intervention have been sustained, including: real-time tracking and replacement of supplies and materials; use of clinical forms for tracking and treatment algorithms; and mentorship visits from the District office for routine system planning.

## Discussion

Across three countries, each with 7 years of implementation experience, project successes included significant increases in data quality across intervention facilities; the integration of data utilization and action-planning in the public sector in Mozambique and Rwanda; an increase from <10% at baseline to >80% of basic clinical protocols being followed in intervention facilities in rural Zambia; and ascension through stage VII on the PIH framework model of data utilization in Zambia, and stage IX in Mozambique and Rwanda – evidenced by the fact they are sustaining data-driven decision-making and scaling-up activities to mentor others in new health areas and geographic regions. All countries included iterative performance measurement and feedback as a core intervention component. There is a rich literature on performance measurement and feedback interventions aimed at improving individual clinical performance in North America and Europe, with meta-analyses showing that these approaches can significantly improve clinical practice and often have the largest effects when baseline performance is low and when supervision structures are strong [33]. The Zambia project represents one of the few measurement-feedback randomized controlled trials focused at the clinician level in a LMIC setting, showing positive results with intensive supervision and investment. The Mozambique and Rwanda projects, however, differ in their application of measurement-feedback loops primarily at higher management levels, with facility, district, and provincial managers being the primary target of data review and QI action planning with feedback cycles.

Through our shared experience, the repeated visualization and presentation of high-quality health data, celebration of change in data protocols and systems, and the identification of QI champions, can all help engender a culture where all staff believe system improvement is possible, and work collaboratively to openly track and engage in QI. Consensus emerged across all countries that the explicit involvement of the Ministry of Health, and collaborative performance review and feedback involving all stakeholders, helped foster a supportive environment focused on collective improvement and culture shifts regarding the importance of using data for decision-making. This is in contrast to the generation of negative feelings or the desire to distance one’s self from system performance which commonly occurs under an external “audit” system.

Common barriers encountered at the planning stage included a long culture of non-use of routine data, a lack of faith in data because of real or perceived problems in data quality, and a lack of belief that change in data quality and/or health system performance was possible. Reflecting on these common barriers, each country team focused on *changing the data use culture* and *ensuring that high-quality data exist*. All teams achieved at least Stage IV or V on our modified Berwick’s data utilization for QI stages (Fig. 2) prior to progressing to the “Do” QI step. Mozambique, for example, achieved stage VI through the collective achievement of the goal of >80% RHIS data concordance, which supported the idea that health system improvement is possible through collective action. All three country interventions began with DQA approaches to improve data analysis and interpretation skills, to introduce data visualization techniques so that high and low performing areas in data quality could be easily identified, as well as to convince stakeholders that rapid improvements in RHIS quality are possible.

A number of challenges were also identified during the “Do” and “Study” periods. In Zambia, although mentorship and supervisory work was designed to be jointly implemented with the district medical officer with an eye towards sustainability, less than half of the visits were completed together. There was consensus across countries that interventions should focus on all members of the health system to avoid the negative effects of staff turnover. A performance review and feedback intervention targeting all health system participants can avoid the potential pitfalls of focusing a highly technical intervention on only a few individuals, such as what was encountered in Zambia, while still maintaining elements of audit-feedback interventions proven efficacious in meta-analyses from higher-income settings [33, 34]. Furthermore, we recommend data-use QI interventions be based on strengthening and using existing RHIS data systems that are Ministry owned and operated in order to have the highest probability for sustainability and long-term changes in data-use cultures [1]. Our findings and consensus coincides with leaders in the field of data-driven QI who suggest that interventions to improve data-use for decision-making prioritize the engagement of data users and producers; iterative DQAs; identification of stakeholder information needs; capacity building in data-use competencies; strengthening data demand and use; along with monitoring, evaluation, and communication of data-use findings [35].

One of the largest challenges for the “Study” and “Act” phases, and an area that requires innovative approaches, is how to conduct *process* and *impact evaluation* of work to increase data-driven QI interventions nested within LMIC health systems. For example, how can we accurately measure that health workers, managers, and policymakers are enacting *data-driven* decision-making rather than changes based on convenience or opinions. There are few structured and validated tools to measure evidence-based decision making in health systems, and what tools exist have primarily been developed and tested in high-income countries for use in measuring individual practitioner’s decision-making [36, 37]. Barriers to promoting evidence-informed decision making from high-income settings have included a lack of incentives, lack of funding, perception that policymakers are not interested in evidence-based practice, and perceived lack of expertise [38–40]. Furthermore, another recent study identified five latent factors of evidence-based decision making, including: capacity for evaluation, expectations and incentives, access to resources, participatory decision-making, and leadership support [41]. A number of these components mirror our observations through implementation experiences across Mozambique, Rwanda, and Zambia; however, future efforts should be undertaken to determine the extent to which psychometric properties of surveys and identified latent factors of evidence-based decision making differ across settings.

Given the necessity for flexibility in concepts and approaches for participatory data-driven QI, thought should be given to how we can create tools and measurement approaches that will allow valid and reliable comparisons of data-driven QI interventions across settings. Future studies and similar interventions must devise implementation measures and easy ways to track successes or failures of action plans and build these performance measures into overall program effectiveness estimation, as well as cumulative learning to optimize performance review and feedback interventions at individual or systems levels in LMICs [34]. However, there is an ongoing struggle between systematizing indicators and approaches that could streamline process and impact evaluation activities, while still providing appropriate flexibility for health system managers to customize QI approaches to their unique settings and respond to rapidly changing data needs, measurement approaches, and treatment guidelines and policies. Staff turnover is another major challenge which impacts continuity of action plan review and QI activities across quarters at the health facility level. Evolving management and supervision priorities through turnover at higher district and provincial levels can further inhibit making longer-term sustainable change. In sum, we believe this is one of the main challenges for the field of implementation science in the coming years – how to accurately evaluate the effects of QI interventions, even randomized ones, which require local adaptations and are inherently tied to local implementation factors using our current statistical modeling or analysis frameworks. Some advocates have called for abandoning rigid protocol-driven randomized study methods, in favor of mixed-methods, or time-series approaches relying on RHIS data [1, 42]; although, it is clear that many in the field still consider the randomized trial to be the “gold standard” in all types of evaluation research, even for QI.

## Conclusions

After a diversity of implementation experience, we have a number of take-away lessons for future data-driven QI activities in LMICs. First, efforts should focus on promoting a culture of data quality and interpretation as a shared responsibility across levels of the health system. To achieve this, we argue that Ministry staff must be explicitly engaged and involved to ensure performance review is seen as collaborative and internally-driven rather than viewed as an external “audit”, as well as to create the space and approval for change, and inevitably, for failure. Second, early in intervention roll-out, intervention leadership should focus on the introduction of DQA methods for improving health data quality. Not only will this help engender a culture shift to believing that rapid change in both data quality and system performance is possible through collective effort, it will ensure confidence in data going forward. Third, relatively “simple” interventions in partnership with Ministry of Health partners, around improving data use, changing the data use culture, and focusing on data-driven performance feedback, mentoring and supervision, and action-planning for *all* health system participants can help avoid pitfalls of investing heavily in a limited set of individuals who may leave their positions, and can help increase intervention sustainability. Last, further work is needed to develop innovative frameworks and models for process and impact evaluation of data-driven QI interventions to help further drive improvements in the science of QI.

## Additional files

Additional file 1: Areas of focus, interventions conducted, and findings of the Rwanda PHIT project to increase data-driven quality improvement. (DOCX 27 kb)

Additional file 2: Before and after comparisons of data completeness on key indicators for patient care amongst 42 primary health clinics undergoing BHOMA intervention from 2011 to 2015. (DOCX 27 kb) (DOCX 26 kb)

## Abbreviations

AHI: African Health Initiative
DDCF: Doris Duke Charitable Foundation
DQA: Data Quality Assessment
EMR: Electronic medical record
IDIs: In-depth interviews
LMICs: Low- and middle income- countries
MESH: Mentoring and Enhanced Supervision at Health Centers program
PDSA: Plan-Do-Study-Act framework
PHIT: Population Health Implementation and Training partnerships
PIH: Partners In Health
QI: Quality improvement
RHIS: Routine health information systems
