# Supplementary material for: Data-driven quality improvement in low-and middle-income country health systems: lessons from seven years of implementation experience across Mozambique, Rwanda, and Zambia
Source: BMC Health Serv Res. 2017 Dec 21;17(Suppl 3):830. doi: 10.1186/s12913-017-2661-x (PMC5763308; doi:10.1186/s12913-017-2661-x)
Supplement: Supplementary file 1 — Areas of focus, interventions conducted, and findings of the Rwanda PHIT project to increase data-driven quality improvement. (DOCX 27 kb) [file 12913_2017_2661_MOESM1_ESM.docx]

| Additional File 1. Areas of focus, interventions conducted, and findings of the Rwanda PHIT project to increase data-driven quality improvement. | | |
| --- | --- | --- |
| **Area of focus** | **Interventions** | **Examples of Results and selected quotes** |
| *Data quality* | Data quality assessment (DQA) with feedback on results.  Training on DQA and data analysis to increase use.  Increase in data feedback at management and district meetings to increase value of more accurate data. Support at the national level to measure and value data quality. | Increase in community health worker data quality (Mitsunaga *et al.* 2013; Gimbel *et al.* 2017) |
| *Data access and use through feedback* | Increase in data feedback at district and other management meetings. | Race to the Top Initiative using feedback of measurement to drive performance based financing to improve targeted indicators (Nahimana *et al.* 2015); Use of data to identify and drive new QI initiatives in neonatal mortality (Magge 2016) and hospital QI (Ingabire), |
| *Culture and skills to utilize data for decision making* | Improved data visualization and timely data feedback linked with mentoring on data-informed decision making. | Effective data-driven allocation of limited resources to improve health facility infrastructure and services (Iyer *et al.* 2015), Neonatal mortality initaitive including quartertly performance measurement review to drive QI (Magge *et al.* 2016) |
| *Culture and skills for QI* | Training on QI including model for improvement and use of performance measurement to identify gaps and track change, establishment of MESH program integrating data use in mentoring for clinical and systems improvement. | Improvement in quality driven by performance measurement-directed mentoring (Anatole *et al.* 2013), hospital-based QI interventions driven and monitored by measurement with feedback (Ingabire *et al.* 2015; Magge *et al. 2016*) |
| *Increasing capacity for implementation research* | Field-based operations research training, establishment of Master’s program at the national school of public health focused on implementation research using routinely available data | Research capacity building supplement paper (Hedt-Gauthier *et al.* 2016). |
