# Supplementary material for: Data-driven quality improvement in low-and middle-income country health systems: lessons from seven years of implementation experience across Mozambique, Rwanda, and Zambia
Source: BMC Health Serv Res. 2017 Dec 21;17(Suppl 3):830. doi: 10.1186/s12913-017-2661-x (PMC5763308; doi:10.1186/s12913-017-2661-x)
Supplement: Supplementary file 2 — Before and after comparisons of data completeness on key indicators for patient care amongst 42 primary health clinics undergoing BHOMA intervention from 2011 to 2015. (DOCX 27 kb) (DOCX 26 kb) [file 12913_2017_2661_MOESM2_ESM.docx]

| Additional File 2. Before and after comparisons of data completeness on key indicators for patient care amongst 42 primary health clinics undergoing BHOMA intervention from 2011-2015. | | | | |
| --- | --- | --- | --- | --- |
| **Indicator** | **Year** | **Proportion complete of 2,000 consultations**  **N (%)** | **95% CI** | **P-Value**  **(comparing 2010 to 2015)** |
| **Temperature** | 2010 | 251 (12.6%) | 11.2-14.1 |  |
|  | 2015 | 1715 (85.9%) | 84.3-87.4 | <0.0001 |
| **Pulse** | 2010 | 2 (0.1%) | 0.02-0.4 |  |
|  | 2015 | 1514 (75.7%) | 73.8-77.5 | <0.0001 |
| **Respiratory rate** | 2010 | 1 (0.05%) | 0.007-35 |  |
|  | 2015 | 1,420 (71.0%) | 69.0-72.9 | <0.0001 |
| **Blood pressure measured** | 2010 | 168 (8.4%) | 7.3-9.7 |  |
|  | 2015 | 1,630 (81.5%) | 79.7-83.1 | <0.0001 |
